# Malignant Insulinoma with Multiple Liver Metastases and Hypercalcitoninemia in a Patient with Type 2 Diabetes Mellitus Presenting as Recurrent Episodes of Diaphoresis due to Severe Hypoglycemia

**DOI:** 10.1155/2020/4239679

**Published:** 2020-01-31

**Authors:** Marco Ciacciarelli, Gianluca Caruso, Marco Rengo, Piero Maceroni, Carmen Misurale, Eleonora D'Armiento, Alessandro Polidoro, Cristina Napoli, Alberto Lombardini, Umberto Ceratti, Ruben Manuel Luciano Colunga Biancatelli, Leonardo Calvosa, Romina Milanese, Sonia Ferri, Teresa Massaro, Andrea Lorusso, Veronica Sorrentino, Vincenzo Petrozza, Luigi Iuliano

**Affiliations:** ^1^Department of Medico-Surgical Sciences and Biotechnologies, Internal Medicine Unit, ICOT Hospital, “Sapienza” University of Rome, Via Franco Faggiana 1668, Latina 04100, Italy; ^2^Department of Medico-Surgical Sciences and Biotechnologies, Pathology Unit, ICOT Hospital, “Sapienza” University of Rome, Via Franco Faggiana 1668, Latina 04100, Italy; ^3^Department of Radiological Sciences, Oncology and Pathology, ICOT Hospital, “Sapienza” University of Rome, Via Franco Faggiana 1668, Latina 04100, Italy

## Abstract

Insulinoma is an insulin-producing pancreatic neuroendocrine tumor that can be malignant in about 10% of cases. Locoregional invasion, lymph node metastases, or remote metastases are the main criteria of malignant insulinoma. Its incidence in patients with pre-existing diabetes mellitus (DM) is exceptionally rare. In this report, we describe a 66-year-old man with long-standing type 2 DM who presented with recurrent episodes of diaphoresis due to severe hypoglycemia despite the withdrawal of insulin therapy, hypercalcitoninemia, and biochemical and radiological findings suggestive of metastatic malignant insulinoma. Unfortunately, after few days of diazoxide treatment, edema, hypotension, oliguria, and water retention were observed, patient's clinical status deteriorated rapidly, and he died in our department from acute renal failure.

## 1. Introduction

Insulinoma is an insulin-producing pancreatic neuroendocrine tumor (PNET) that can be malignant in about 10% of cases [1]. Locoregional invasion, lymph node metastases, or remote metastases are the main criteria of malignant insulinoma. The incidence of insulinoma, especially the malignant type, in patients with diabetes mellitus (DM) is extremely rare, and the diagnosis can be challenging due to concomitant glucose-lowering drugs, mistaken as the cause of hypoglycemia. Here, we report the case of a 66-year-old man with a medical history of long-standing type 2 DM diagnosed with malignant metastatic insulinoma, presented as recurrent episodes of diaphoresis. Furthermore, we discuss its relevance within the contest of PNETs and similar cases described in the literature, to aware physician of this, yet rare but dramatic and challenging disease that could affect the diabetic population.

## 2. Case

A 66-year-old man was admitted to our internal medicine department for recurrent episodes of severe hypoglycemia. Type 2 DM was diagnosed when he was 31 years old and initially treated with oral antihyperglycemic agents. The patient discontinued oral antidiabetic agents and started insulin therapy 4 years before admission. At that time, he was treated with long-acting insulin glargine and insulin aspart. This insulin regimen was switched to insulin degludec/liraglutide 3 months before admission. While he was on insulin degludec/liraglutide, his home blood glucose monitoring ranged from 110 to 70 mg/dL. Five days before admission, while on insulin degludec/liraglutide in a dose of 18 IU daily, the patient started having recurrent episodes of diaphoresis. During those episodes, usually in the early morning before breakfast, his blood glucose levels were below 50 mg/dL. The patient suffered from several further episodes despite insulin dosage tapering and even after discontinuation. His past medical history was notable for heart failure with reduced ejection fraction, acute coronary syndrome, and osteomyelitis of the left foot. His family medical history included atrial fibrillation and DM. In addition to insulin, his therapy consisted in acetylsalicylic acid, bisoprolol, furosemide, ramipril, and atorvastatin. He denied oral antihyperglycemic agents. He had no known drug allergies, he was a former smoker, and he did not drink alcohol or use illicit drugs.

On admission, the patient's blood pressure was 130/80 mmHg, heart rate was 71 beats per minute, respiratory rate was 14 breaths per minute, and oxygen saturation was 98%, while he was breathing ambient air. The patient's body mass index was 26.7 kg/m^2^ (height: 173 cm; weight: 80 kg), and the physical examination was unremarkable. Laboratory data on admission showed glucose 50 mg/dl, HbA1c 50 mmol/mol, normocytic anemia (hemoglobin 11.9 g/dL, hematocrit 34.8%, and mean corpuscular volume 85 fl), a normal white cell and platelet count, aspartate aminotransferase (AST) 87 IU/L (normal range: 17–59), alanine aminotransferase (ALT) 62 IU/l (normal range: 21–72), gamma glutamyl-transferase 545 IU/l (normal range: 15–73), alkaline phosphatase 177 IU/l (normal range: 38–126), total bilirubin 0.47 mg/dL (normal range: 0.20–1.30), lactate dehydrogenase 1021 IU/L (normal range: 313–618), albumin 3.5 g/dL (normal range: 3.6–5.5), INR 1.18, C-reactive protein 7.5 mg/dl (normal range: 0-1), erythrocyte sedimentation rate 90 mm/h (normal range: 0–15), fibrinogen 621 mg/dl (normal range: 170–410), iron 37 *μ*g/dl (normal range: 49–181), and ferritin 776 ng/ml (normal range: 20–325). Viral markers for hepatitis B and C were negative.

Further blood tests were ordered to rule out endogenous hyperinsulinism: C-peptide was 4.2 ng/mL (normal range: 0.5–3), insulin 89 *μ*IU/mL (normal range: 5–25), while the patient's glucose level was 40 mg/dL, and sulfonylurea screening test was negative, confirming endogenous hyperinsulinism. Markers of neuroendocrine tumors were severely increased: chromogranin A (CgA) was 1790 ng/ml (normal range: 0–100), and neuron-specific enolase (NSE) was 458 ng/ml (normal range: 0–11).

**Figure 1:**
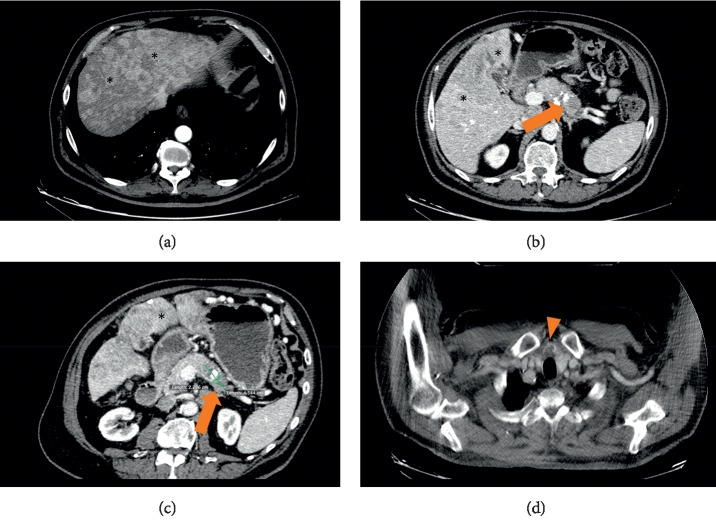
CT images (a)–(c) show multiple hypervascular liver metastases (*∗*) and a focal solid lesion of the pancreatic tail (arrow). A thyroid nodule (arrow head) was also detected (d).

**Figure 2:**
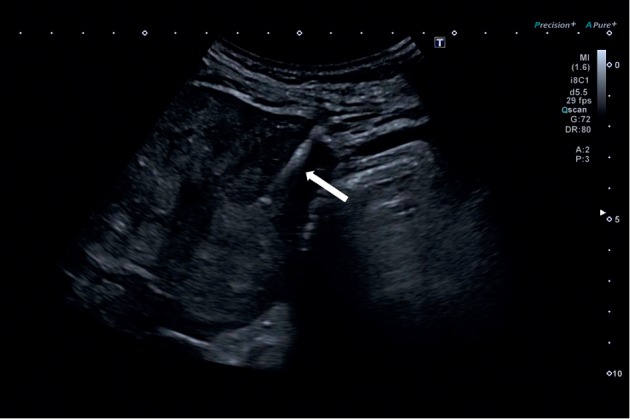
Ultrasound-guided percutaneous biopsies of the lesion located in the liver segments II-III. White arrow shows the needle penetrating in the subglissonian isoechoic/hypoechoic solid nodule with irregular margins (maximum diameter: 2.2 cm).

**Figure 3:**
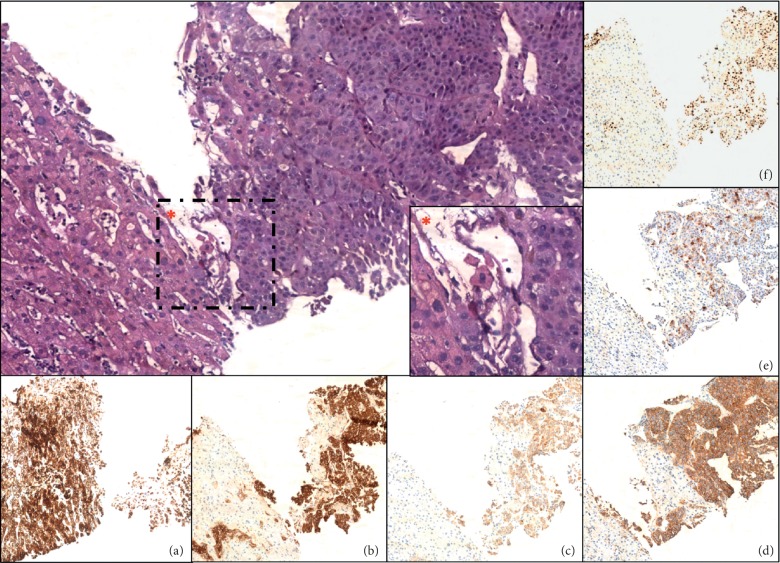
Poorly differentiated neuroendocrine carcinoma metastatic to the liver (H&E, magnification: 10x). The *∗* box represents a detailed view of neoplasia (H&E, magnification: 40x). Immunohistochemical staining for CAM5.2 (a), synaptophysin (b), chromogranin A (c), CD56 (d), insulin (e), and Ki-67 proliferation index (f) (magnification: 10x).

**Figure 4:**
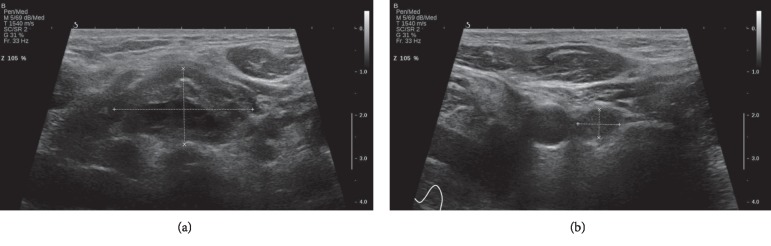
Thyroid ultrasound. Weakly hypoechoic solid nodule (2.8 × 3.2 × 1.3 cm) with a central cystic component and regular margins located in the isthmus (a). Markedly hypoechoic solid nodule (0.9 × 0.9 × 0.6 cm) adjacent to the lower pole of the left lobe (b).

Given the high suspicion of insulinoma and the altered liver function tests on admission, a full-body CT scan was ordered. CT scan showed a 4.3 × 2.2 cm pancreatic tail mass containing calcifications with multiple regional lymphadenopathies (Figure 1). Multiple liver secondary lesions were found: the largest ones measuring 12 and 11 cm, respectively, in II-III and IVb-V segments. No lungs, bone, and brain metastases were detected. Additionally, a thyroid nodule of 2.6 cm was incidentally found. Hepatic metastases were highly suspected to be secondary to a malignant insulinoma, so ultrasound-guided percutaneous biopsies of the lesion in the II-III segments were performed (Figure 2). Histological findings of four core biopsies consisted of hepatocytes infiltrated by a poorly differentiated and focally necrotic proliferating tissue of medium-sized atypical epithelial cells with solid growth pattern (Figure 3). The cancer cells showed hyperchromatic nuclei with “salt and pepper” chromatin markedly Ki-67 positive, high mitotic index (10 figures × 10 HPF), and scant cytoplasm. Immunohistochemistry studies revealed the neuroendocrine nature and the partial insulin secreting activity of the lesion; CgA, synaptophysin, CD56, and CAM5.2 were diffusely positive; there was patchy (<5%) insulin expression. Ki-67 index was 40–50. These findings were consistent with the diagnosis of metastatic, poorly differentiated (G3), neuroendocrine carcinoma with morphology and immunophenotype favoring a pancreatic primary tumor. Thyroid ultrasound (Figure 4) showed a weakly hypoechoic solid nodule (2.8 × 3.2 × 1.3 cm) with a central cystic component and regular margins located in the isthmus and a markedly hypoechoic solid nodule (0.9 × 0.9 × 0.6 cm) adjacent to the lower pole of the left lobe. Calcifications and intranodular flow were absent in both nodules, and regional lymph nodes were not enlarged. The results of thyroid function tests were within normal limits (TSH 0.7 mUI/L, normal range: 0.46–4.68), as well as were parathormone (PTH) (80.1 pg/mL, normal range: 13.6–85.8), calcium (8.76 mg/dL, normal range: 8.4–10.2), and phosphorus (4.32 mg/dL, normal range: 2.5–4.5). Serum calcitonin was slightly raised (30 pg/mL, normal range: 0–6), but immunohistochemical staining of the liver lesions was negative for such markers.

The patient's hospital stay was characterized by different episodes of severe hypoglycemia (as low as 18 mg/dL) occurring especially at night and in the early morning, manifesting only with diaphoresis. During those episodes, the patient never lost consciousness or developed seizures and even never complained of confusion, amnesia, weakness, diplopia, blurred vision, palpitations, or hunger. In order to reduce the hypoglycemic events, we started a symptomatic treatment with diazoxide and IV infusion of 10% dextrose, while waiting for histopathological confirmation of the disease. Unfortunately, due to the severity of this malignancy, after 8 days of treatment edema, hypotension, oliguria, and weight gain of 10 kg were observed; the patient's clinical status deteriorated rapidly, and 3 days later, the patient died from acute renal failure. Postmortem examination was not carried out according to his family's request.

## 3. Discussion

PNETs are rare neuroendocrine malignancies that count only for the 2% of total pancreatic tumors. Their incidence is around 1 case per 100,000 people, and only about 10% of them are classified as functional, as they release hormones or peptides that cause specific symptoms and signs [2]. With an estimated incidence of 4 cases per 1 million person-years [3], insulinomas and insulin-producing tumors, are the most common functional PNETs, and unlike other PNETs, which are malignant in 50–100% of cases, they are malignant only in about 10% of cases [4].

Locoregional invasion, lymph node metastases, or remote metastases are the main criteria of malignant insulinoma. Most patients with malignant insulinoma have lymph node or liver metastases [5–7], and only rare metastases in other sites such as bones [8, 9], lungs [10], brain [11], ovaries [12, 13], and gallbladder [14]. Liver metastases at presentation count for almost 60% of the patients with PNETs [2].

In this report, we described a malignant insulinoma with multiple liver metastases in a 66-year-old man with long-standing type 2 DM. Although benign insulinoma occurs rarely in patients with pre-existing DM [15–17], malignant insulinoma in this population seems to be even rarer, and only sparse cases are described in the literature, in both type 2 [18–23] and type 1 DM [12, 24, 25]. However, despite the rare occurrence of insulinoma in diabetic population, clinicians should be aware of this possible differential diagnosis when managing diabetic patients with recurrent episodes of symptomatic hypoglycemia despite tapering and withdrawal of glucose-lowering drugs. We searched the PubMed database, and to the best of our knowledge, we collected the clinical, pathologic, and biochemical data of all previously reported cases of malignant insulinoma in patients with DM (Table 1). As reported by Yu et al. [26], short hypoglycemic symptoms duration before diagnosis of tumor seems to be common in patients with malignant insulinoma (median duration: 30 days). Furthermore, as shown in the same study, high proinsulin and CgA levels and a large proinsulin/insulin molar ratio should raise the suspicion of malignant insulinoma in patients with hyperinsulinemic hypoglycemia. In our case, similarly, hypoglycemic symptoms duration before diagnosis of malignant insulinoma was short (20 days), and CgA levels were markedly elevated (1790 ng/ml). Unfortunately, we could not measure proinsulin levels and calculate proinsulin/insulin molar ratio due to laboratory limitations.

Insulinoma is a pancreatic islet cell tumor which may be associated with multiple endocrine neoplasia type 1 (MEN-1). MEN-1 diagnosis was excluded because of age, a negative family medical history, and the absence of any clinical features of the most common endocrine and nonendocrine tumors associated with this rare syndrome. Primary hyperparathyroidism is commonly associated with MEN-1, and at least 90% of patients develop it by the age of 50 years. The absence of history of osteoporosis or nephrolithiasis and normal serum calcium and PTH levels led us to rule out primary hyperparathyroidism. Since our patient had no symptoms or signs of a pituitary tumor, we did not order pituitary hormones (other than TSH) and pituitary MRI to rule out a pituitary tumor.

Metastatic insulinoma in our patient was extremely aggressive, and only progressively increased doses of diazoxide, up to 500 mg per day, were able to avoid severe hypoglycemic episodes. Unfortunately, as reported in other cases [19], our patient rapidly developed edema, hypotension, oliguria, and water retention secondary to diazoxide toxicity and died from acute renal failure. First-line chemotherapy regimen for poorly differentiated pancreatic neuroendocrine carcinoma consists with a combination of etoposide and either cisplatin or carboplatin. Unfortunately, when we obtained the report of histological analysis of liver biopsy, our patient was hemodynamically unstable and his renal function has worsened significantly, so we were not able to start chemotherapy with traditional regimen. Everolimus is an mTOR inhibitor with hyperglycemic and antitumoral effects indicated in treatment of malignant insulinoma. Given its toxicity, it is used as third-line treatment of recurrent episodes of hypoglycemia after failure of diazoxide and somatostatin analogs. The antitumoral effect of everolimus has been shown to improve progression-free survival of patients with well-differentiated PNETs. However, the antitumor efficacy of everolimus in poorly differentiated pancreatic neuroendocrine carcinoma remains to be determined.

Interestingly, our patient displayed increased serum calcitonin levels (30 pg/ml, normal range 0–6). Calcitonin is a peptide hormone secreted by C cells of the thyroid that acts as a physiologic antagonist to PTH, lowering blood calcium levels. Despite hypercalcitoninemia is a typical marker of medullary thyroid carcinoma (MTC), calcitonin can be found increased in several conditions: after exhausting physical activity, because of interfering drugs, in thyroid and non-thyroid diseases. These conditions should be always ruled out in cases with basal calcitonin values between 10 and 100 pg/mL [27], as observed in our patient.

He was sedentary, and at the time of blood sample analysis, he was not taking interfering drugs (e.g., omeprazole, glucocorticoids, or beta-blockers). In fact, bisoprolol was discontinued on admission in the attempt of increasing the adrenergic warning symptoms of hypoglycemia. Although glucagon-like peptide 1 analogs such as liraglutide are well known to cause slightly increases of calcitonin, we rejected this hypothesis because our patient discontinued liraglutide 22 days before calcitonin measurement.

Follicular and papillary thyroid carcinomas are thyroid diseases that may be associated to slightly raised calcitonin levels. Although the two thyroid nodules of our patient appeared benign and did not show any signs of malignancy (e.g., calcifications, intranodular flow, taller-than-wide shape, irregular margins, local invasion of surrounding structures, suspicious neck lymph nodes), we could not completely rule out any type of thyroid carcinoma, including MTC, as fine needle aspiration was not carried out due to severe worsening of the patient's clinical status. However, it is unlikely that ultrasound thyroid findings paired with thyroid malignancies and two different neuroendocrine tumors such as MTC and malignant insulinoma developed simultaneously.

Hypercalcitoninemia may also occur in nonthyroid diseases such as chronic renal failure, hyperparathyroidism-related hypercalcemia, and hypergastrinemia. Our patient had normal renal function, normal calcium levels, and no clinical features of conditions commonly associated with hypergastrinemia such as atrophic gastritis and gastrinoma; therefore, serum gastrin levels were not measured.

Hypercalcitoninemia due to ectopic calcitonin secretion by several types of PNETs, including insulinoma, has been described [28–33]; therefore, we measured calcitonin levels even if our patient had no family history of MTC or clinical and laboratory findings consistent with hyperparathyroidism or pheochromocytoma. Moreover, hypercalcitoninemia due to calcitonin-producing insulinoma has been documented in patients with benign thyroid nodules [31, 32].

Since a specific endocrine syndrome related to hypercalcitoninemia has not been identified yet, calcitonin-producing PNETs are currently classified as nonfunctional, unless they secrete “eutopic” biologically active hormones (insulin, glucagon, somatostatin, and pancreatic polypeptide). Interestingly, according to Uccella et al. [33], calcitonin expression is not related to either a more aggressive behavior or a worse prognosis, so it seems to not identify a separate subset of PNETs. The immunohistochemical detection of calcitonin in cells from pancreatic mass and the normalization of its value after tumor resection are important findings in confirming the diagnosis of calcitonin-producing insulinoma [29, 31, 32]. Due to the history of heart failure with reduced ejection fraction and the high suspicion of liver metastases, we performed a less-invasive diagnostic procedure such as liver biopsy rather than endoscopic ultrasound-guided biopsies of the pancreatic mass, as successfully reported in similar cases [5–7]. To the best of our knowledge, the few cases of calcitonin-producing insulinoma reported in the literature were not associated to metastases in liver or in other sites. In our case, immunohistochemistry of the liver lesion was negative for calcitonin; however, it should be noted that immunostaining of hepatic lesions secondary to calcitonin-producing PNETs may be negative for calcitonin probably because metastases can dedifferentiate and lose their secretory activity [34].

Despite the diagnosis of malignant insulinoma in our patient was made with biopsy of liver metastasis rather than biopsy of the pancreatic mass, in the absence of other apparent causes of hypercalcitoninemia, we hypothesized that cells of the pancreatic malignancy were able to produce calcitonin with following elevation of this serum marker.

## 4. Conclusion

In conclusion, although very rare, malignant insulinoma can occur in patients with both type 2 and type 1 DM. Most patients with malignant insulinoma have lymph node or liver metastases at presentation. Malignant insulinoma with liver metastases should always be considered as a cause of recurrent episodes of symptomatic hypoglycemia in diabetic patients with altered liver function tests, especially when severe hypoglycemia persists after withdrawal of glucose-lowering agents.

Furthermore, this case underlines that hypercalcitoninemia in patients with thyroid nodules is not necessarily due to MTC. Several physiologic and pathologic conditions other than MTC have been associated with increased calcitonin levels, including calcitonin-producing PNETs. Since the majority of PNETs are malignant, nonfunctional and hence represent a diagnostic challenge for physicians, all patients with raised calcitonin levels and thyroid nodules not clearly suggestive of MTC should be submitted to a careful investigation in order to rule out a calcitonin-producing PNET, a rare but recognized cause of hypercalcitoninemia.

## Figures and Tables

**Table 1:** Clinical, pathologic, and biochemical data of reported cases of malignant insulinoma in patients with diabetes mellitus.

Authors	Year	DM type	Age, sex	Site	Size (cm)	Metastases	Hypoglycemia symptoms	Insulin (*μ*UI/ml)	C-peptide (ng/ml)	CgA (ng/ml)	Thyroid disease	Calcitonin (pg/ml)
Svartberg [12]	1996	1	33, F	Tail	Not reported	Liver Ovaries	Unconsciousness	86	8.4	Not reported	No	Not reported
Siraji [18]	2006	2	74, F	Tail, head	7.3 × 5.7 3 × 3 3.1 × 1.8	Liver	Diaphoresis Palpitations Confusion Seizures	39 (G: 28)	5.2	Not reported	No	Not reported
Schmitt [19]	2008	2	79, F	Body, tail	Not reported	Liver	Hunger Dizziness Tiredness	74 (G: 34)	9.7	↑ (404)	Hypothyroidism	Normal (<3)
FerrerGarcìa [20]	2011	2	78, M	Head	4.7 × 3	Liver	Not reported	23 (G: 35) (F)	4.6	↑ (834)	No	Not reported
Abbasakoor [21]	2011	2	67, F	Body	2.6 × 2 5-6	Liver Lymph nodes	Unconsciousness Anxiety Diaphoresis	91 (G: 19) (F)	8.2	Not reported	No	Not reported
Ademoglu [22]	2012	2	45, F	Head	0.8 × 0.7 0.9 × 1 0.7 × 0.7	Liver Lymph nodes	Confusion Diaphoresis	114 (G: 35) (F)	4.8	Not reported	No	Not reported
Lablanche [24]	2015	1	31, M	Head	6.3 × 5.6	Lymph nodes	Not reported	18 (G: 37) (F)	7.8	Not reported	Hashimoto thyroiditis	Not reported
Gjelberg [25]	2017	1	43, F	Tail	11 × 7	Liver Lymph nodes	Not reported	9 (G: 45) (F)	1.6	↑ (323)	Hypothyroidism	Normal
Our patient	2019	2	66, M	Tail	4.3 × 2.2	Liver	Diaphoresis	89 (G:40)	4.2	↑ (1790)	Thyroid nodules	↑ (30)

G: glucose (mg/dl); F: fasting.
